# Calcified double J stent removed at 10 years: a case report

**DOI:** 10.11604/pamj.2022.41.94.30722

**Published:** 2022-02-02

**Authors:** Ramzi Mejri, Kays Chaker, Mokhtar Bibi, Sami Ben Rhouma, Yassine Nouira

**Affiliations:** 1 Departement d’Urologie, Centre Hospitalier Universitaire Mongi Slim la Marsa, Tunis, Tunisie,; 2 Departement d’Urologie, Hospital la Rabta, Tunis, Tunisie

**Keywords:** Double j stent, calcification, incrustation stent, case report

## Abstract

In our current practice, the use of JJ probes has become extremely frequent. However, incrustation and fragmentation of JJ leads are still relatively common and sometimes complicate removal. A 61-year-old woman with a history of hysterectomy ten years ago, she had a double J endo-ureteral stent for preoperative identification of the right ureter. The patient has forgotten the double J stent. She currently has right lower back pain and urinary tract symptoms of urinary. Uroscan revealed a very important right hydronephrosis, a fully calcified right double J stent with a calcification of 6 cm at the level of the lower loop. She had, at first, a cystotomy allowing the extraction of the lower part of the calcified stent and secondly a pyelotomy to extract the rest of the double J stent. The operative follow-up was simple. The use of a double J probe to divert the urinary tract is an effective and generally well tolerated technique. Regular monitoring prevents complications.

## Introduction

Since the appearance of the double J ureteral endoprosthesis in 1978 [1], its use has become extremely common and the composition of the probes has evolved. However, the encrustation and fragmentation of the probes remain frequent situations, sometimes complicating the removal. In the majority of cases, this phenomenon is minimal and the ablation is done without difficulty.

## Patient and observation

**Patient information:** a 61-year-old woman with a history of hysterectomy ten years ago, she had a double J endo-ureteral catheter for preoperative identification of the right ureter. The patient has forgotten the double J stent. She currently has right lower back pain and urinary tract symptoms of urinary frequency and urinary burns.

**Clinical findings:** when examined, she was weak and had vital signs within normal limits: blood pressure 110/55mmHg; temperature 36.8 C; pulse 90/min; respiratory rate 22/min. the physical examination is strictly normal.

**Diagnostic assessment:** the bacteriological examination of urine was negative. Uroscan revealed a very important right hydronephrosis, a fully calcified right double J stent with a calcification of 6 cm at the level of the lower loop.

**Figure 1:**
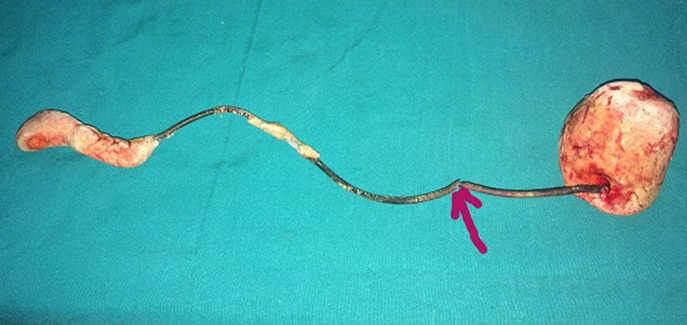
appearance of the probe after removal

**Therapeutic intervention:** she had, at first, a cystotomy allowing the extraction of the lower part of the calcified double J stent and secondly a pyelotomy to extract the rest of the double J stent (Figure 1).

**Follow-up and outcomes:** the operative follow-up was simple. The radiological control at 6 months by a computed tomography (CT) scan returned normal.

**Patient perspective:** during the time she was hospitalized and after the treatment, the patient was delighted with the care she received and was optimistic about the outcome of her condition.

**Informed consent:** the patient was informed about the case report, why her case was peculiar and the authors' interest in publishing her case. She willingly gave informed consent to allow the authors to use her photos for this case report. Informed consent was obtained from the client for us to use the pictures.

## Discussion

Encrustation of ureteral stents is a well-known phenomenon that can be easily treated with early treatment. However, a severe encrustation constitutes a major complication because of the renal lesions it causes and its removal is sometimes heavy and risky. The factors favoring encrustation are well documented. They are multifactorial. The duration of drainage is reported by the majority of authors as being a determining factor [1]. Bacterial colonization or urinary tract infection, pregnancy, chemotherapy, is commonly reported [2]. Almost 75.5% of the probes had become encrusted within 6 months and 42.8% within 4 months. The mean indwelling time was 5.6 months [3]. Many techniques have been described for removing encrusted stents [4]. However, Bukkapatnam obtained an excellent result using the holmium laser with a rigid and / or flexible ureteroscope since they treated 12 embedded probes in 10 patients in a single step [5]. Open surgery is rarely reported, but we have used it because there is a large kidney stone associated.

## Conclusion

The use of a double J catheter to divert the urinary tract is a safe and well-tolerated method, although it is not free from complications, which is why we must make patients aware of the need to undergo periodic controls and instruct them about possible symptoms and or signs that may indicate alterations in the position and or status of the stent.
